# Social media marketing and digital influence for visitor flow management in sustainable heritage tourism

**DOI:** 10.1038/s41598-025-28555-9

**Published:** 2025-12-11

**Authors:** Chenyi Shen

**Affiliations:** Faculty of Arts and Humanities, University of Southampton, Southampton, UK

**Keywords:** Business and management, Business and management, Cultural and media studies, Cultural and media studies

## Abstract

Chinese cultural heritage sites face severe overtourism challenges, necessitating innovative management approaches. This study examines associations between social media marketing and visitor flow management and its potential contribution to sustainable heritage tourism. Through empirical analysis of five major Chinese sites (Forbidden City, Temple of Heaven, Giant Wild Goose Pagoda, Terracotta Warriors, and Mogao Grottoes), combining 507 visitor questionnaires with 100,000 social media posts, this research employs PLS-SEM modeling to examine dual pathways of association. Findings reveal that 78.3% of tourists rely on social media information for itinerary planning, with 67.4% willing to adjust visit timing based on real-time crowd information. Path analysis identifies a cognitive-informational pathway where perceived usefulness (β = 0.452) and information timeliness (β = 0.387) are directly associated with tourist response willingness while also showing indirect associations via decision confidence (β = 0.312 and β = 0.287, respectively), alongside an affective-social pathway where information source credibility (β = 0.268) and platform interaction (β = 0.195) are associated with response willingness via emotional resonance (β = 0.183). Past travel experience negatively moderates these associations (β= − 0.156). These findings support the potential of digital strategies to advance SDG 8.9, 11.4, and 12.b targets, offering implications for cultural heritage management.

**Supplementary Information:**

The online version contains supplementary material available at 10.1038/s41598-025-28555-9.

## Introduction

Overtourism has emerged as a common challenge facing cultural heritage sites globally, with the dramatic increase in visitor numbers threatening not only the physical integrity of heritage assets but also the quality of life for local communities and visitor experiences^1,2^. Traditional visitor management measures such as capacity restrictions and price increases, while demonstrating certain effectiveness, have revealed numerous limitations in practice and struggle to achieve the dual objectives of visitor satisfaction and heritage conservation^3^. The rapid development of social media has provided new approaches to addressing this dilemma, as digital platforms profoundly influence tourists’ destination choices and behavioral patterns through information dissemination and opinion leader effects. Particularly in China, domestic social platforms including Weibo, TikTok, and Rednote have become primary channels for tourists to obtain travel information, with their digital influence reshaping visitor flow patterns at cultural heritage sites^4^. In recent years, as China advances its green economy transformation, sustainable tourism has been prioritized as a key development direction, with precision marketing through social media to guide spatial-temporal visitor distribution and optimize tourist experiences proving to be an effective strategy for promoting sustainable utilization of cultural heritage^5^. This soft management approach based on digital influence can both alleviate pressure on popular sites and enhance visitor satisfaction, opening new pathways for achieving harmonious coexistence between cultural heritage conservation and tourism development.

Despite the enormous potential social media has demonstrated in visitor flow management, its mechanisms of action and impact pathways require further theoretical exploration. Understanding how digital influence translates into tourist behavioral change necessitates comprehensive analysis from multidisciplinary theoretical perspectives. The Theory of Planned Behavior provides a fundamental framework for analyzing this transformation process, positing that attitudes, subjective norms, and perceived behavioral control collectively determine behavioral intentions. In the social media context, platform information influences tourists’ perceptions of crowding levels, judgments about others’ behaviors, and assessments of travel convenience, thereby altering their spatial-temporal choice decisions^6^. Concurrently, Uses and Gratifications Theory reveals tourists’ intrinsic motivations for actively utilizing social media, where tourists are no longer passive information recipients but actively seek real-time crowd information, promotional offers, and travel guides based on specific needs. This agency creates conditions for implementing precise guidance strategies^7^. From a macro perspective, the Tourism Area Life Cycle Theory illustrates that cultural heritage sites at different development stages face differentiated management challenges, with overcrowding problems particularly acute at mature-stage heritage sites, necessitating innovative intervention measures to delay decline trends^8^. The recently emerging metacognition theory explains at a deeper level how social media stimulates tourists’ self-reflection and sense of responsibility, prompting them to balance personal enjoyment with collective interests. This cognitive-level transformation is key to achieving sustainable tourism^9^.

Based on the aforementioned theoretical framework, this study aims to investigate the specific mechanisms and optimization pathways of social media marketing in visitor flow management at cultural heritage sites. While existing research recognizes that overtourism requires solutions from a sustainability perspective^10^ and has preliminarily explored the relationship between social media and visitor flows^11^, critical questions remain insufficiently examined, including how digital influence may be associated with tourist behavioral change, the differential effects of various information sources, and the construction of optimal information strategies. Therefore, this study focuses on three core research questions: How does social media information influence tourists’ spatial-temporal distribution decisions—specifically, how do tourists’ timing choices and site selection behaviors change after receiving different types of digital information? What differences exist in influence, credibility, and conversion effectiveness between traffic-directing information published by official accounts versus Key Opinion Leaders (KOLs), and what implications do these differences hold for marketing strategy formulation? Which types of information content, publication timing, and dissemination methods most effectively guide visitor flows to achieve peak-shaving and valley-filling management objectives? Based on these research questions, the study’s objectives include: empirically evaluating the actual impact of social media marketing on the spatial-temporal distribution of visitor flows; identifying key factors influencing tourist responses to social media information and their impact pathways; and providing scientific evidence and practical guidance for achieving coordination between heritage conservation and tourism development.

This study holds significant value for advancing both the theory and practice of sustainable heritage tourism. From a theoretical contribution perspective, the research integrates social influence theory with digital marketing theory to construct a visitor flow management framework applicable to cultural heritage sites, extending the application boundaries of traditional tourism management theory in the digital era. Additionally, the study reveals the underlying mechanisms through which social media may relate to tourist behavior, enriching the theoretical foundations of tourist behavior research and providing new perspectives for understanding tourism decision-making processes in the digital age. From a practical standpoint, the research outcomes provide heritage site managers with data-driven decision-making tools, facilitating the development of more precise and effective social media marketing strategies. By identifying the differential effects of various information types and publishing entities, the study provides scientific evidence for optimizing resource allocation and enhancing management efficiency. More importantly, this research directly responds to the United Nations World Tourism Organization’s initiatives on managing urban tourism growth^12^ and makes positive contributions to achieving the United Nations Sustainable Development Goals: promoting sustainable tourism development through optimizing visitor flow distribution (SDG 8.9), strengthening cultural heritage protection by alleviating visitor pressure (SDG 11.4), and improving sustainable tourism assessment tools through establishing digital monitoring systems (SDG 12.b). China’s innovative practices as a major world heritage nation not only provide replicable management models for developing countries but also contribute Chinese solutions for digital transformation to global cultural heritage conservation.

## Literature review

### Cultural heritage overtourism research

The overtourism phenomenon at cultural heritage sites has attracted widespread academic attention in recent years, with research spanning from conceptual definitions and impact assessments to management strategies, forming a relatively comprehensive body of knowledge. Overtourism is defined as a phenomenon where visitor numbers exceed destination carrying capacity, resulting in declining quality of life for local residents, deteriorating visitor experiences, and degradation of environmental and cultural resources^13^. While this concept remains somewhat contested, its core focus—the negative impacts of tourism development—has become widely accepted^14^. Historic city centers, as important carriers of cultural heritage, face particular pressures and challenges, with the high concentration of tourist activities not only causing physical space congestion but also producing cumulative damage to historic buildings and cultural relics, threatening the authenticity and integrity of cultural heritage^15^. Research indicates that overtourism at cultural heritage sites exhibits distinct spatial-temporal concentration characteristics, with popular attractions bearing visitor pressure far exceeding design capacity during specific periods, while surrounding areas and off-peak periods experience underutilization of resources^14^. This uneven distribution exacerbates management difficulties, as traditional volume control measures struggle to effectively address structural imbalances^13^. Furthermore, the non-renewable nature of cultural heritage makes the consequences of overtourism particularly severe, as damage once inflicted is often irreversible, requiring managers to adopt preventive rather than remedial management strategies. While existing research has established a foundation for understanding the complexity of overtourism, further exploration is needed regarding innovation in management models within the digital context.

### Social media and tourism decision-making

Social media has emerged as a critical factor influencing contemporary tourism decision-making, with its role permeating the entire tourism behavior process. Early research primarily focused on the instrumental role of social media in tourism information search, finding that tourists increasingly rely on social platforms to obtain destination information, compare tourism products, and formulate itineraries^16^. As research has progressed, scholars have recognized that social media’s influence extends far beyond information transmission functions, profoundly transforming tourists’ decision-making patterns through user-generated content, social interaction, and emotional resonance^17^. Persuasion mechanisms on social media manifest through multidimensional influence pathways, including informational and normative influence, where tourists not only acquire practical information from content shared by others but also form expectations and preferences about destinations through observing others’ behaviors. This socialization process significantly affects their final destination choices and behavioral intentions^18^. Tourism experience sharing behaviors further amplify social media’s influence, as tourists posting photos, videos, and reviews not only document personal experiences but become information sources influencing others’ decisions, creating a continuous cycle of influence^19^. Particularly noteworthy is how social media has transformed the traditional linear decision-making process, enabling tourists to obtain real-time destination information, dynamically adjust itinerary arrangements, and even change original plans based on social media recommendations during their journey. This immediacy and interactivity provides new intervention opportunities and methods for visitor flow management, making it possible to guide tourist behavior through digital channels.

### Visitor flow management strategies

The profound influence of social media on tourist decision-making has prompted scholars to reexamine traditional flow management strategies and explore innovative management models for the digital era. Early visitor flow management research primarily focused on physical space regulation, employing interventions such as price adjustments, capacity limitations, and route design through analysis of spatial-temporal distribution patterns of visitor flows. While these static management methods can control total visitor volume to some extent, they struggle to address dynamically changing tourist demands and sudden congestion issues^20^. The integration of digital technology has brought revolutionary changes to visitor flow management, with modern management systems emphasizing the combination of real-time monitoring, intelligent prediction, and dynamic guidance. Through big data analytics and artificial intelligence technologies, managers can accurately grasp visitor flow trends and respond promptly^21^. Spatial econometric research has further revealed network effects in visitor flows, where information releases and marketing activities at one attraction produce spillover effects on visitor distribution throughout an entire region. These findings provide scientific evidence for formulating collaborative management strategies and achieving balanced regional visitor flow distribution^22^. Notably, soft management strategies based on information guidance are becoming mainstream trends. By delivering real-time crowd information, personalized tour suggestions, and incentive measures to tourists, these strategies guide autonomous itinerary adjustments. This approach not only avoids negative experiences associated with mandatory measures but also fully leverages tourist agency. The widespread application of social media platforms provides ideal channels for implementing such soft management strategies, enabling precise and personalized flow control.

Individual differences in tourism experience and expertise also warrant consideration, as prior knowledge has been shown to moderate information processing and decision-making patterns. Experienced tourists, having accumulated destination knowledge and travel skills, tend to rely more on internal cues and less on external information sources compared to novice tourists^23,24^. This expertise effect suggests that the influence of social media marketing on tourist behavioral responses may vary across different tourist segments, with implications for developing targeted communication strategies.

### Sustainable tourism and SDGs

The emergence of soft management strategies reflects the tourism industry’s deepening understanding of sustainable development principles, which aligns closely with the United Nations Sustainable Development Goals (SDGs). Discussions on sustainable urban tourism have transcended the singular focus on overtourism, shifting toward more comprehensive sustainability assessment frameworks encompassing multiple dimensions including economic benefits, social equity, environmental protection, and cultural preservation^25^. Information technology plays a pivotal role in promoting sustainable tourism development, with digital tools not only enhancing resource utilization efficiency and reducing environmental impacts but also strengthening stakeholder participation and facilitating knowledge sharing and innovation^26^. However, a significant gap exists between academia and practice in advancing sustainable tourism, with substantial research remaining at the theoretical discussion level, lacking operational implementation plans and impact assessments. This phenomenon of “much talk, little action” constrains the achievement of sustainable tourism objectives^27^. Emerging technologies such as artificial intelligence present new opportunities for achieving SDGs, enabling more precise balancing of tourism development and resource conservation through intelligent prediction, personalized recommendations, and automated management. Nevertheless, these technologies also introduce potential risks including data privacy concerns and algorithmic bias, necessitating careful evaluation and regulatory guidance^28^. Cultural heritage tourism, as a crucial domain for sustainable development, directly relates to the achievement of specific targets including SDG 8.9 (promoting sustainable tourism), SDG 11.4 (protecting cultural heritage), and SDG 12.b (developing monitoring tools). The innovative application of social media marketing strategies provides feasible pathways for attaining these objectives.

### Research gaps

Despite substantial achievements in existing research on overtourism, social media influence, flow management, and sustainable development, significant deficiencies remain in effectively integrating these elements to address practical problems at cultural heritage sites. While scholars have recognized the potential of social media and big data technologies in understanding tourist mobility patterns^29^, most studies remain at the level of phenomenon description and correlation analysis, lacking exploration of the causal mechanisms through which social media information translates into tourist behavioral change. Existing literature rarely distinguishes the differential impacts of various types of social media accounts (official versus KOL), overlooking the moderating effects of information source characteristics on tourist responses. Regarding empirical research, most studies employ single data sources or cross-sectional data, failing to capture the dynamic processes and long-term effects of social media influence. More critically, localized research focusing on Chinese cultural heritage sites remains relatively scarce. Given China’s unique social media ecosystem and cultural context, the applicability of foreign experiences requires validation. Furthermore, existing research lacks systematic frameworks that integrate social media marketing strategies with specific SDG targets, failing to provide actionable guidance for management practice. These research gaps highlight the necessity and urgency of conducting the present study.

### Research hypotheses

The preceding literature review reveals several critical gaps that this study aims to address. While existing research has established that social media influences tourist decision-making, the specific mechanisms through which different information characteristics affect behavioral responses in heritage tourism contexts remain underexplored. Drawing upon the Elaboration Likelihood Model^30,31^, we propose that social media information operates through dual pathways: a cognitive-informational pathway where information quality attributes (perceived usefulness and timeliness) directly influence response willingness and indirectly operate through decision confidence; and an affective-social pathway where source characteristics (credibility and platform interaction) indirectly influence response willingness through emotional resonance. Furthermore, recognizing that information processing varies with tourist expertise^23,24^, we propose that tourism experience moderates these relationships. Building on Source Credibility Theory^32^ and Technology Acceptance Model perspectives^33^, along with empirical findings on tourist information search behavior^23,24^, we formulate the following hypotheses to test these theoretical propositions in the context of cultural heritage visitor flow management.

H1: Social media information characteristics are positively associated with tourists’ response willingness through dual pathways (cognitive-informational and affective-social).

H1a: Perceived usefulness and information timeliness positively influence tourist response willingness directly and indirectly through decision confidence.

H1b: Information source credibility and platform interaction positively influence tourist response willingness indirectly through emotional resonance.

H2: Past travel experience negatively moderates the relationship between social media information and tourist response willingness.

## Materials and methods

### Research design overview

This study employs a mixed-methods research design, integrating quantitative and qualitative analytical approaches to comprehensively reveal the mechanisms through which social media marketing influences visitor flows at cultural heritage sites. The research design follows a progressive logic of “status diagnosis → mechanism exploration → effect validation,” divided into four interrelated phases. The first phase involves objective status assessment (January 2023–December 2024), establishing a research baseline and identifying key issues through large-scale collection of social media data, official visitor flow statistics, and heritage monitoring reports. This phase focuses on analyzing the matching relationship between social media information dissemination characteristics and the spatial-temporal distribution of visitor flows, revealing structural imbalances between information supply and demand. The second phase comprises visitor perception validation (April–May and October–November 2024), conducting field questionnaire surveys at five case study sites to gain in-depth understanding of visitors’ exposure to different types of social media information, credibility perception, and behavioral responses, compensating for the inability of secondary data to reflect subjective perceptions. This study received ethical approval exemption from the University of Southampton’s Ethics and Research Governance Committee, as it involved low-risk, anonymous survey research conducted in accordance with the Declaration of Helsinki, with voluntary participation and no collection of personally identifiable information. The third phase involves causal relationship modeling, employing Partial Least Squares Structural Equation Modeling (PLS-SEM) to examine the impact pathways of social media characteristics on tourist behavior. PLS-SEM was selected over covariance-based SEM for this exploratory study examining emerging social media influence mechanisms in heritage tourism. Preliminary Shapiro–Wilk tests revealed that several key constructs violated normality assumptions (Information Timeliness: W = 0.947, *p* < 0.01; Platform Interaction: W = 0.953, *p* < 0.01), making PLS-SEM’s distribution-free approach more appropriate. This method is particularly suitable for exploratory research, capable of simultaneously handling measurement and structural models while effectively identifying complex relationships between variables^34^. This multi-phase, multi-method triangulation strategy ensures the robustness of research findings, with mutual corroboration between different data sources and analytical methods enhancing the credibility of conclusions. The overall research design embodies a complete research chain from phenomenon description to mechanism explanation and practical application.

### Case selection and sample determination

Case selection followed theoretical sampling principles, comprehensively considering multiple criteria including heritage type representativeness, visitor pressure levels, and social media activity. Five typical Chinese cultural heritage sites were ultimately selected as research subjects: the Forbidden City (palace complex, annual visitor flow 19 million), Temple of Heaven (altar architecture, annual visitor flow 16 million), Terracotta Warriors (underground site, annual visitor flow 9 million), Giant Wild Goose Pagoda (Buddhist architecture, annual visitor flow 8 million), and Mogao Grottoes (grotto art, annual visitor flow 2.6 million with strict capacity controls implemented). These cases encompass different types of cultural heritage, varying levels of visitor pressure, and different stages of digital management development, ensuring the generalizability of research findings. The social media account sample includes 15 official accounts (5 sites × 3 platforms) and 50 relevant KOL accounts (with over 1 million followers and engagement rates exceeding 5%). These thresholds were consistently applied across all five heritage sites, with 10 KOLs identified per site based on content relevance and posting frequency (minimum 2 posts/month about the respective site during the study period). The questionnaire survey sample was initially planned at 100 valid responses per site (120 distributed per site accounting for invalid responses, totaling 600 questionnaires). During actual field implementation, 653 questionnaires were distributed across the five sites to compensate for lower-than-anticipated response rates at certain locations and to replace incomplete surveys. After excluding 146 incomplete or inconsistent responses, 507 valid questionnaires were retained, yielding an effective response rate of 77.6%. Sample size adequacy was verified through a priori power analysis using G*Power 3.1. For a structural equation model with 8 latent variables, medium effect size (f^2^=0.15), α = 0.05, and desired power = 0.80, the minimum required sample size was 160. The final sample size exceeds the minimum requirements for PLS-SEM analysis^35^. The sample temporally covers both peak and off-peak seasons and spatially encompasses site entrances, core areas, and exits, ensuring sample representativeness and diversity.

### Data collection methods

Social media data collection employed a multi-source data fusion strategy to ensure research comprehensiveness and accuracy. Social media data were automatically collected through Python web scraping programs, covering three major platforms: Weibo, TikTok, and Rednote. The collection captured six complementary data dimensions for each post: (1) textual content (captions, descriptions, comments); (2) visual content (images and videos); (3) temporal metadata (publication timestamps, posting time of day); (4) engagement metrics (repost counts, comment counts, like counts); (5) geographical tags (check-in locations, GPS coordinates when available); and (6) account attributes (official accounts vs. KOL accounts, follower counts). This multi-dimensional approach enabled comprehensive profiling of social media discourse around heritage tourism, capturing not only what was said (textual content) but also how it was communicated (visual elements), when it was published (temporal patterns), how it resonated with audiences (engagement metrics), and where it was generated (spatial information). The data collection period spanned from January 2023 to December 2024, yielding over 100,000 relevant posts with associated metadata. Each post was assigned a unique identifier and stored in a structured database with normalized field formats to facilitate subsequent multi-modal integration and analysis^36^.

Questionnaire surveys employed on-site intercept methods, randomly selecting tourists at different site locations (entrance 40%, core area 40%, exit 20%). Survey content encompassed social media usage habits, information exposure, decision-influencing factors, and behavioral change intentions. This design enabled comprehensive understanding of social media influence throughout the entire tourism process^37^. Visitor flow data were sourced from official site statistics, real-time monitoring systems, and third-party platforms (Gaode, Baidu Heat Maps), obtaining crowd data at different temporal granularities (hourly/daily/weekly/monthly). Heritage monitoring data were obtained through reviewing annual reports from heritage conservation departments and academic literature at each site, including key parameters such as structural deformation, mural fading rates, and microenvironmental indicators.

### Data analysis methods

Data analysis employed a multi-level, multi-method comprehensive analytical framework. Social media text data were subjected to content mining using Latent Dirichlet Allocation (LDA) topic modeling implemented via Python’s gensim library. The optimal number of topics was determined through systematic evaluation of model coherence scores and perplexity across K = 5 to K = 15 topics, with K = 8 yielding the highest coherence score (Cv = 0.52) and acceptable perplexity (487.3). Hyperparameters were set as follows: α = 0.1 (document-topic density, promoting sparse topic distributions per document), β = 0.01 (topic-word density, promoting focused word distributions per topic), 1000 iterations with convergence threshold of 0.001. The eight identified topics represented distinct thematic dimensions including crowd conditions, cultural experiences, practical information, visual aesthetics, service quality, accessibility, seasonal recommendations, and heritage conservation concerns. Topic distributions for each post were used as features in subsequent cross-modal analysis^38^. Sentiment analysis was conducted using a fine-tuned BERT (Bidirectional Encoder Representations from Transformers) model based on the bert-base-chinese pretrained architecture from Hugging Face’s transformers library (version 4.30.2). A training dataset of 3000 social media posts (1200 positive, 1200 negative, 600 neutral) was manually annotated by two independent coders (inter-rater reliability Cohen’s κ = 0.83). The BERT model was fine-tuned for three epochs with learning rate 2e−5, batch size 16, and maximum sequence length 128 tokens. On a held-out test set (*n* = 500), the model achieved 87.3% accuracy, with F1-scores of 0.89 (positive), 0.84 (negative), and 0.78 (neutral). Sentiment scores were integrated with other modality features to assess emotional dimensions of social media discourse^38^.

The integration of heterogeneous social media data followed a systematic fusion framework to standardize diverse data modalities into a unified analytical structure. Raw data from three platforms (Weibo, TikTok, Rednote) encompassed six data types: textual content, visual content (images and videos), temporal metadata (publication timestamps), engagement metrics (reposts, comments, likes), geographical tags, and account attributes. To enable cross-modal analysis, each modality was transformed into standardized numerical features: textual content was processed through LDA topic modeling (K = 8 topics) and BERT sentiment analysis to generate topic distribution vectors and sentiment polarity scores; visual content was manually coded (10% sample, *n* = 10,247) and then automatically classified into scene categories (e.g., crowded scenes, architecture) using trained image classification models; temporal data were normalized to Beijing Time and encoded as categorical time segments; engagement metrics were min-max normalized (0–1 scale) to create platform-independent interaction intensity scores; and geographical tags were geocoded to heritage site identifiers. This standardization process converted multi-modal raw data into a unified feature vector representation for each post, enabling systematic cross-modal pattern analysis.

Quantitative data analysis centered on Partial Least Squares Structural Equation Modeling (PLS-SEM) to examine tourist questionnaire data (*n* = 507). The analysis assessed how tourists’ perceptions of social media information characteristics were associated with their behavioral response willingness, as measured through self-reported survey items. The study operationalized eight latent constructs through multi-item scales adapted from validated instruments. Independent variables included: Information Source Credibility (ISC, 4 items)—the degree to which tourists perceive social media sources as trustworthy and reliable, adapted from Chung and Han^18^ and Xiang and Gretzel^16^; Perceived Usefulness (PU, 5 items)— tourists’ assessment of how social media information aids travel decisions, adapted from Kim and Fesenmaier^19^ and Leung et al.^17^; Information Timeliness (IT, 4 items)—the promptness of information delivery, adapted from Vu et al.^36^ and Fotis et al.^37^; Platform Interaction (PI, 4 items)—engagement intensity with content, adapted from Whiting and Williams^39^ and Liu et al.^40^; and Emotional Resonance (ER, 4 items)—emotional connections evoked by content, adapted from Liu et al.^40^ and Chung and Han^18^. Mediating variable comprised Decision Confidence (DC, 4 items) tourists’ certainty in their travel decisions based on social media information^6,37^. Dependent variables comprised Tourist Response Willingness (TRW, 5 items)—intention to adjust behavior, adapted from Ajzen^6^ and Chung and Han^18^. Past Travel Experience (PTE, 3 items) served as a moderator variable. All constructs used 5-point Likert scales (1 = Strongly Disagree to 5 = Strongly Agree). Complete measurement items and detailed operational definitions are provided in Appendix A.

Logistic regression analysis was utilized to predict the probability of tourists adjusting behavior based on social media information, quantifying the influence of different factors on binary decision outcomes through odds ratios (OR). Two-way ANOVA tested interaction effects between seasonal factors and information types on tourist responses, identifying significant differences under various condition combinations. Measurement model assessment followed established guidelines^34,41^. Internal consistency reliability was evaluated using Cronbach’s alpha (α) and composite reliability (CR), with acceptable thresholds of 0.70. Convergent validity was assessed via average variance extracted (AVE), requiring values above 0.50. Factor loadings for individual items were examined, with values exceeding 0.70 considered satisfactory. Discriminant validity was examined using the heterotrait-monotrait ratio (HTMT), with values below 0.85 indicating adequate discriminant validity^41^. Multicollinearity was checked through variance inflation factors (VIF), with values below 3.0 considered acceptable. Detailed reliability, validity, and collinearity assessment results are presented in Tables S4–S7 (Appendix B). All statistical analyses were completed using Stata 17 and SmartPLS 3.0 software, with significance level set at 0.05.

Given that all constructs were measured through self-reported questionnaire data, we assessed potential common method bias (CMB) using Harman’s single-factor test. All measurement items were entered into an unrotated exploratory factor analysis.

Word cloud visualization was employed to complement the structured PLS-SEM analysis by capturing emergent themes from open-ended responses that might not be fully captured by pre-defined scales. While the questionnaire measured predetermined constructs, tourists’ spontaneous concerns—extracted through TF-IDF weighting and LDA topic modeling—provide ecological validity checks and reveal unanticipated priorities. This mixed-method triangulation strengthens construct validity and ensures the measurement model reflects actual tourist decision-making factors rather than researcher-imposed categories.

### Research ethics and data management

This study strictly adhered to academic research ethical standards, fully protecting participant rights and data security. Prior to questionnaire survey implementation, all participants were informed of the research objectives, data usage, and confidentiality measures, with surveys conducted only after obtaining written informed consent. Participants retained the right to withdraw from the study at any time without consequence. Purposive sampling in qualitative research followed principles of scientific rigor and representativeness, ensuring samples adequately reflected the characteristics of the research population^42^. Social media data collection was limited to publicly published content, strictly complying with platform terms of service and relevant laws and regulations, without involving user privacy information. All personal identifying information underwent anonymization before data processing, with coding systems replacing actual identity markers.

### Software tools

The study employed various specialized software to complete data collection, processing, and analysis tasks. During the data collection phase, Python 3.9 programming environment was utilized, with Selenium and BeautifulSoup libraries implementing automated social media data scraping, while Pandas library was used for data cleaning and preprocessing. Text analysis employed Python’s jieba segmentation tool for processing Chinese text, gensim library for implementing LDA topic modeling, and transformers library for calling BERT models to conduct sentiment analysis. Statistical analysis primarily utilized Stata 17 for descriptive statistics, regression analysis, with its robust panel data processing capabilities particularly suited to this study’s multi-timepoint data structure^43^. SmartPLS 3.0 was employed to construct and test PLS-SEM models, evaluating complex relationships between variables. Visualization tools included Python’s matplotlib and seaborn libraries for generating statistical charts, and Tableau for creating interactive data dashboards to facilitate presentation of spatial-temporal distribution characteristics of visitor flows.

## Results

### Analysis of social media information and tourist flow status

Analysis of social media data from the five case study sites during January 2023 to December 2024 reveals significant differentiation in information dissemination strategies across sites. Table 1 presents a comparison of key social media operation indicators for each site.

**Table 1:** Comparison of social media operation characteristics at five cultural heritage sites (2023–2024).

Site	Daily posts (average)	Heritage knowledge content (%)	Interactive content (%)	Visual display content (%)	Official account engagement rate	KOL account engagement rate
Forbidden City	2.8	43%	25%	32%	3.1%	9.2%
Giant Wild Goose Pagoda	2.3	18%	37%	45%	2.5%	10.3%
Terracotta Warriors	2.1	35%	28%	37%	2.2%	8.1%
Temple of Heaven	1.8	38%	30%	32%	1.9%	7.6%
Mogao Grottoes	1.5	67%*	15%	18%	1.8%	7.3%

*Note: 67% of Mogao Grottoes content consists of reservation reminders and visiting guidelines.

**Fig. 1:**
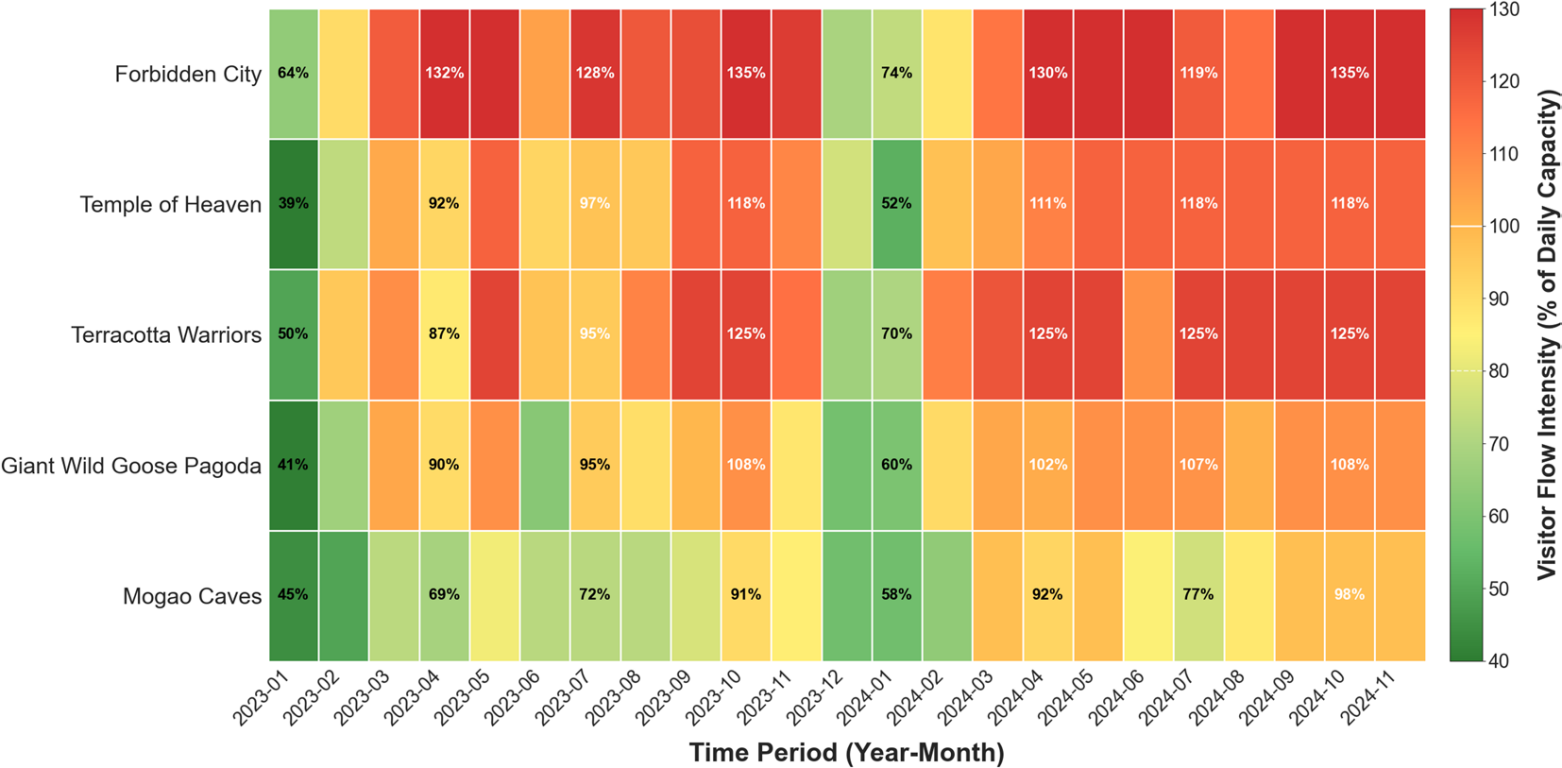
Spatial-temporal distribution heat map of tourist flows (2023–2024).

Significant mismatches exist between the spatial-temporal distribution characteristics of tourist flows and social media information publication timing. As illustrated in the heat map in Fig. 1, visitor flows demonstrate concentrated distribution patterns across temporal and spatial dimensions. Regarding intraday distribution, visitor flows exhibit a typical “M-shaped” double-peak pattern, with the first peak forming between 10 and 11 a.m. when visitor density reaches 2.6 times the daily average, and the second peak occurring between 2 and 3 p.m. with density reaching 2.8 times the daily average. A brief decline occurs between peaks (12 − 1 p.m.), primarily due to visitors temporarily leaving core areas for lunch. Weekly distribution shows weekend (Saturday and Sunday) visitor flows averaging 65% higher than weekdays, with Saturday reaching peak levels. Seasonal distribution indicates that peak season (April–October) accounts for 73.8% of annual visitation, with daily average visitor flows during May Day and National Day Golden Weeks reaching 4.2 times off-season levels.

**Fig. 2:**
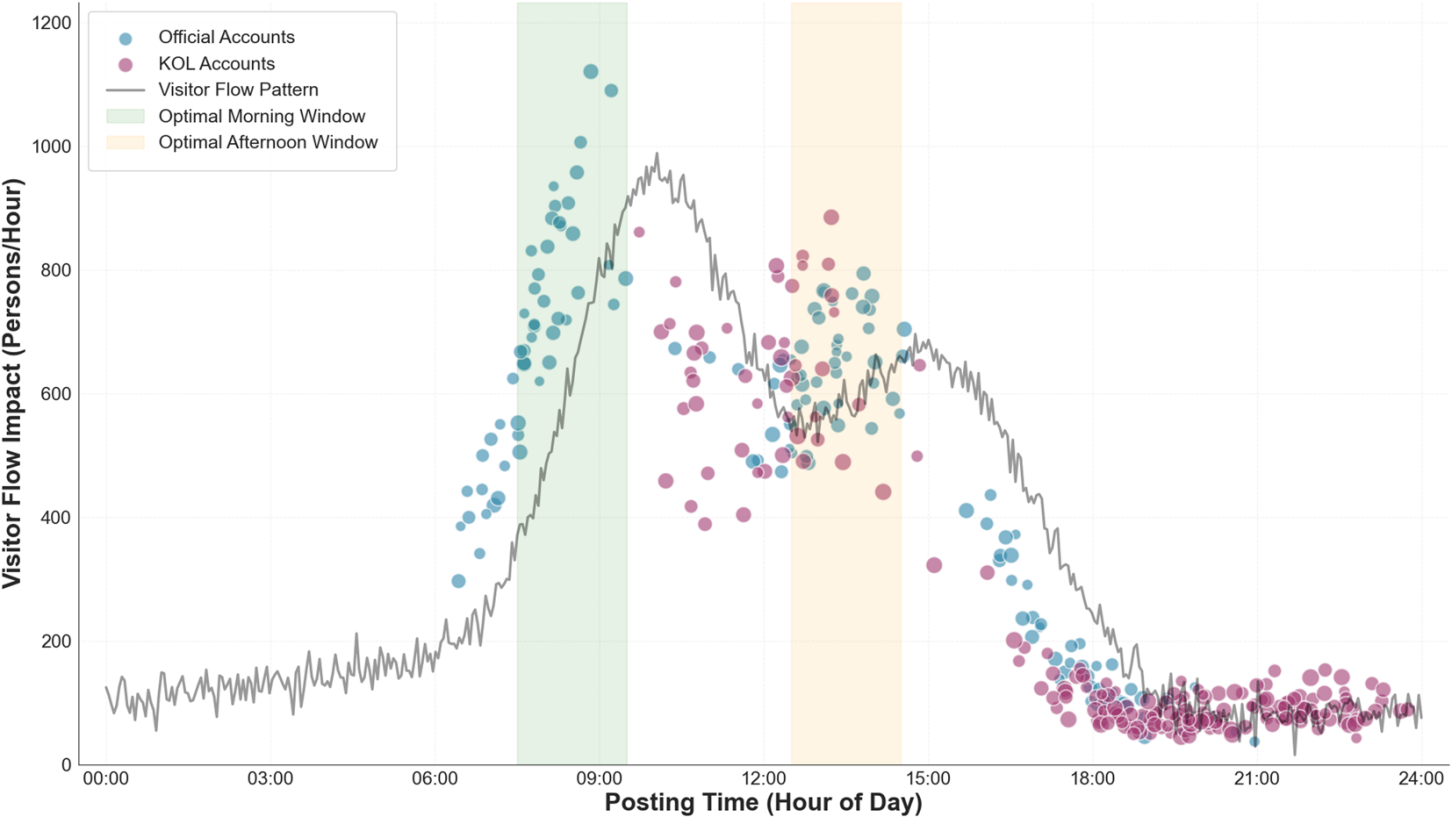
Scatter plot of social media information publication timing and visitor flow peak alignment.

However, as shown in the scatter plot in Fig. 2, severe temporal mismatches exist between social media information publication times and visitor flow peaks. Comparative analysis of 100,000 social media posts against actual visitor flow data reveals that: 65% of crowd alert information is published after peaks have already formed, with an average delay of 3.2 h, completely nullifying warning and diversion functions; only 12% of information is published more than 2 h in advance, genuinely serving preventive guidance functions; 23% of information publication timing basically synchronizes with peak periods (within ± 30 min), demonstrating good timeliness but limited guidance effectiveness. This lag in information dissemination is associated with reduced effectiveness of social media’s role in visitor flow management and may relate to diminished visitor experience due to untimely information. Primary causes of this mismatch include: lack of real-time monitoring and rapid response mechanisms in site management departments, cumbersome official account publication processes resulting in poor timeliness, and insufficient predictive capacity for visitor flow peaks.

### Visitor-side validation of social media influence

While “Analysis of social media information and tourist flow status” revealed objective patterns in social media information dissemination and temporal mismatches with visitor flows through platform data analysis, understanding how tourists actually perceive, process, and respond to such information requires direct investigation of visitor perspectives. Questionnaire survey results reveal the actual response characteristics of tourists to social media information and its influence mechanisms on tourism decision-making. Descriptive statistical analysis results are shown in Table S1. Among 507 valid respondents (653 questionnaires distributed in total, 146 incomplete questionnaires excluded, effective response rate 77.6%), 78.3% indicated they actively search for destination-related social media information before traveling. Table S2 presents platform usage proportions, with Rednote (42.5%) and TikTok (38.7%) emerging as the primary information acquisition channels, while Weibo usage has declined to 18.8%. As shown in Table S3, chi-square test results indicate that age structure is significantly associated with social media usage preferences (χ^2^ = 156.34, *p* < 0.001), with younger age groups demonstrating substantially higher social media information dependence. The 18–25 age group shows the highest dependence rate at 92.0%, followed closely by the 26–35 age group at 86.1%, while those aged 56 and above show only 31.6%, presenting distinct intergenerational differences with a large effect size .Independent samples t-test analysis of trust differences between information sources, with results shown in Table 2, reveals that official account information credibility scores 4.12 (SD = 0.68), significantly higher than KOL accounts at 3.68 (SD = 0.82, t = 12.47, *p* < 0.001). However, paired samples t-test indicates the latter performs more prominently in influencing tourist emotional cognition and stimulating travel motivation, with emotional resonance reaching 4.23 (t = − 8.91, *p* < 0.001).

**Table 2:** Comparison of credibility and emotional resonance scores between different information sources.

Evaluation dimension	Official accounts	KOL accounts	Difference test
Credibility score	4.12 (SD = 0.68)	3.68 (SD = 0.82)	t = 12.47, *p* < 0.001
Emotional resonance	3.45 (SD = 0.71)	4.23 (SD = 0.65)	t = − 8.91, *p* < 0.001
Information accuracy	4.31 (SD = 0.59)	3.52 (SD = 0.88)	t = 14.23, *p* < 0.001
Content attractiveness	3.28 (SD = 0.76)	4.15 (SD = 0.69)	t = − 10.56, *p* < 0.001

Scores based on 5-point Likert scale (1 = very low, 5 = very high); SD = standard deviation.

### Relationship model between social media data and tourist choices

Prior to examining structural relationships, the measurement model was rigorously evaluated to ensure psychometric soundness. As detailed in Table S4, all constructs demonstrated excellent internal consistency reliability, with Cronbach’s alpha values ranging from 0.794 (Past Travel Experience) to 0.908 (Tourist Response Willingness), and composite reliability (CR) values ranging from 0.879 to 0.932, all substantially exceeding the 0.70 threshold. Convergent validity was confirmed, with average variance extracted (AVE) values ranging from 0.654 (Platform Interaction) to 0.733 (Tourist Response Willingness), all surpassing the required 0.50 minimum. Factor loading analysis (Table S5) revealed that all measurement items loaded strongly on their respective constructs, with loadings ranging from 0.776 to 0.879, well above the 0.70 criterion. Cross-loadings were consistently lower than primary loadings, providing additional evidence of construct validity. Discriminant validity was established through the heterotrait-monotrait ratio (HTMT) assessment (Table S6), with all inter-construct HTMT values below 0.85, indicating adequate construct distinctiveness. Harman’s single-factor test was conducted to assess potential common method bias. Exploratory factor analysis revealed that the first factor accounted for 32.4% of the total variance, well below the 50% threshold suggested by Podsakoff and Organ^44^. Furthermore, multicollinearity diagnostics (Table S7) showed all variance inflation factors (VIF) below 2.5, well within the acceptable threshold of 3.0, confirming the absence of problematic multicollinearity in the structural model. These comprehensive assessments provide strong evidence for the reliability, validity, and statistical adequacy of the measurement model, establishing a robust foundation for subsequent structural path analysis.

Logistic regression analysis results indicate that 67.4% of respondents would adjust their visiting time based on real-time crowding information (OR = 2.31, 95% CI 1.86–2.87), with 35.2% willing to advance or delay their trip by 1–2 h to avoid peak periods. Acceptance of alternative site recommendations remains relatively low, with only 28.9% of tourists indicating they would consider changing their original destination. Two-way ANOVA reveals significant seasonal effects in the attractiveness of promotional information (F = 45.67, *p* < 0.001), with 54.3% of tourists choosing to travel due to promotional activities during off-peak seasons, compared to only 21.7% during peak seasons.

**Fig. 3:**
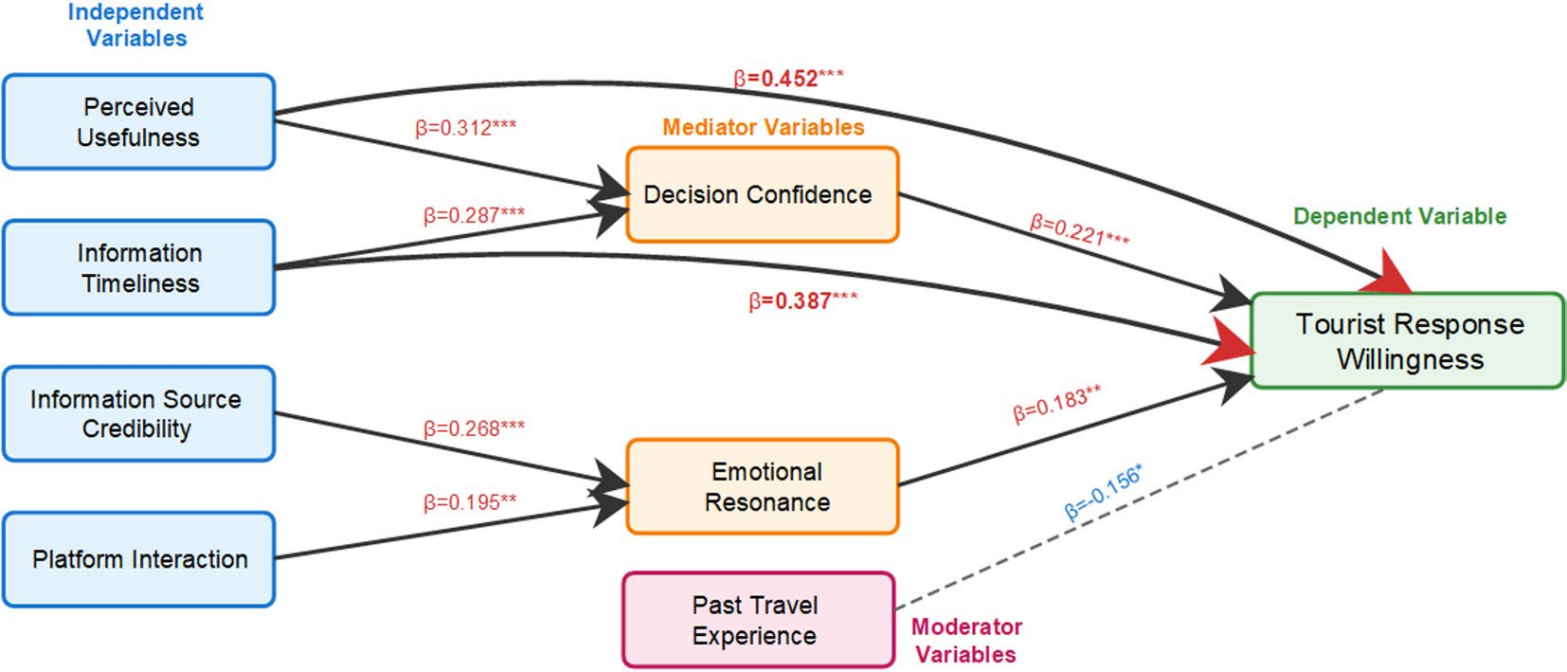
Path analysis diagram of factors associated with tourists’ response willingness to social media information. ***p < 0.001, **p < 0.01, *p < 0.05. Path coefficients are standardized β values from PLS-SEM with 5000 bootstrap samples. Measurement model details (factor loadings, reliability, validity assessments) are provided in Tables S4–S7.

Following the confirmation of measurement model adequacy (see Tables S4–S7 in Appendix B for complete psychometric assessments), the structural model was evaluated to test hypothesized relationships. As illustrated in Fig. 3, path analysis using Partial Least Squares Structural Equation Modeling (PLS-SEM) with 5000 bootstrap samples reveals that perceived usefulness (β = 0.452, *p* < 0.001, 95% CI [0.389, 0.515], f^2^ = 0.281) and information timeliness (β = 0.387, *p* < 0.001, 95% CI [0.328, 0.446], f^2^ = 0.196) exert the strongest direct effects on tourist response willingness, representing large effect sizes according to Cohen’s criteria (f^2^ > 0.15). These two information quality attributes also significantly influence decision confidence (β = 0.312, *p* < 0.001, 95% CI: [0.256, 0.368], f^2^ = 0.134 and β = 0.287, *p* < 0.001, 95% CI [0.231, 0.343], f^2^ = 0.115, respectively), which in turn positively affects response willingness (β = 0.221, *p* < 0.001, 95% CI: [0.165, 0.277], f^2^ = 0.068), demonstrating a significant mediating pathway through the cognitive-informational mechanism. Additionally, information source credibility (β = 0.268, *p* < 0.001, 95% CI [0.212, 0.324], f^2^ = 0.092) and platform interaction (β = 0.195, *p* < 0.01, 95% CI [0.139, 0.251], f^2^ = 0.051) indirectly influence tourist response willingness through the mediating role of emotional resonance (β = 0.183, *p* < 0.01, 95% CI [0.127, 0.239], f^2^ = 0.044), confirming the affective-social pathway. Notably, past travel experience demonstrates a significant negative moderating effect (β= − 0.156, *p* < 0.05, 95% CI [− 0.212, − 0.100], ΔR^2^ = 0.024), indicating that experienced tourists are less likely to adjust their behaviors based on social media information, preferring instead to rely on their own judgment and prior knowledge when planning heritage site visits. Effect size analysis indicates that the cognitive-informational pathway (through decision confidence) exerts substantially stronger influence than the affective-social pathway (through emotional resonance), with the former demonstrating large to medium effects (f^2^ = 0.134–0.281) and the latter showing small to medium effects (f^2^ = 0.044–0.092). The overall model explains 61.8% of the variance in tourist response willingness (R^2^ = 0.618), indicating substantial predictive power.

In summary, the structural model provides full support for all proposed hypotheses. H1a is confirmed through significant direct and indirect effects of perceived usefulness and information timeliness via decision confidence. H1b is supported with information source credibility and platform interaction influencing response willingness through emotional resonance. H2 receives empirical support, confirming the negative moderating role of past travel experience.

### Key factors associated with tourist responses

**Fig. 4:**
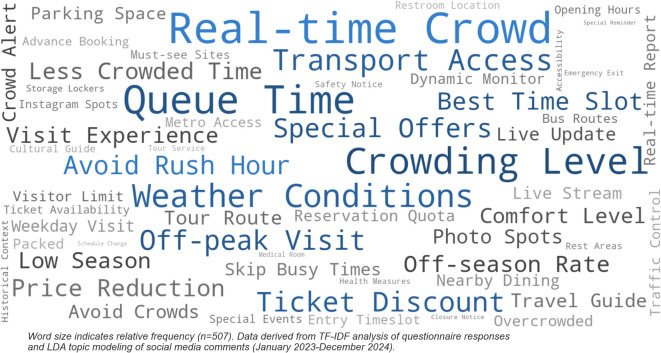
Word cloud of key factors influencing tourist travel decisions.

Through text mining analysis of open-ended questions in 507 valid questionnaires, combined with natural language processing of social media comment data, this study identifies key factors associated with tourists’ responses to social media information and subsequent travel plan adjustments. Applying TF-IDF (Term Frequency-Inverse Document Frequency) algorithms to calculate weights of information elements of tourist concern, and extracting latent semantic features through LDA topic modeling, a word cloud reflecting tourists’ core concerns was generated (Fig. 4).

Word frequency analysis results show that “real-time crowds” (frequency 18.7%), “queue time” (16.2%), and “crowding level” (14.5%) emerge as the three core elements of greatest tourist concern, with this real-time information directly influencing travel timing decisions for 62.3% of respondents. Semantic network analysis further reveals association patterns between keywords, with “ticket discounts” forming strong associations with terms such as “off-season” and “off-peak” (correlation coefficient *r* = 0.73), indicating that price incentive mechanisms play important roles in guiding tourists’ temporal choices. Notably, while “weather conditions” (11.8%) and “transport convenience” (10.2%) show relatively lower frequencies, they demonstrate strong predictive power in multivariate logistic regression analysis (OR = 1.87, *p* < 0.01), particularly showing significant influence on long-distance tourist groups.

BERT model sentiment analysis results indicate that information containing avoidance terms such as “avoid,” “evade,” and “stagger” achieves higher interaction rates (average likes increased by 43.2%), reflecting tourists’ strong aversion to negative crowding experiences. Comparison of vocabulary distribution differences between official and KOL content reveals that KOLs tend to use vivid expressions such as “packed like sardines” and “bursting with crowds” (accounting for 27.3%), while official accounts prefer professional terminology such as “visitor flow” and “carrying capacity” (accounting for 41.5%). These expressive differences partially explain the differential influence between the two information source types. Deep semantic analysis also identifies the implicit dimension of “experience quality,” which, though rarely mentioned directly, is reflected through related vocabulary such as “comfortable,” “leisurely,” and “enjoyable,” with cumulative influence weight reaching 0.21.

## Discussion

The effectiveness of social media marketing can be explained through Uses and Gratifications Theory, whereby tourists actively seek social media information to satisfy diverse needs including information acquisition and social interaction^39^. Survey results show that 78.3% of tourists rely on social media for itinerary planning, with 67.4% adjusting visiting times based on real-time crowd information. Social media also promotes tourism consumption by stimulating “social media envy” emotions^40^, explaining why KOL content achieves higher emotional resonance (4.23) than credibility scores (3.68). Fundamental differences exist between official accounts and KOL marketing strategies: 67% of official account content comprises crowd alerts and visiting guidelines, with visitor flow management as the core objective; whereas KOLs pursue engagement rate maximization (averaging 8.7% versus 2.3% for official accounts), enhancing personal commercial value through vivid expressions and visual impact. These goal-oriented differences determine their functional positioning—official accounts fulfill public service functions while KOLs are driven by influence monetization.

The findings of this study form complementary insights with existing literature. Early research suggested that social media exacerbated overtourism problems, as exemplified by the Barcelona case where Instagram promotion led to visitor surges at specific attractions^45^. However, this study reveals social media’s bidirectional regulatory potential—through real-time information dissemination and differentiated marketing strategies, it can effectively guide spatial-temporal visitor distribution. This finding aligns with Falk and Hagsten’s perspective, who noted that while visitor flows have increased in the Instagram era, digital tools simultaneously provide management opportunities^46^. The key lies in proactive utilization rather than reactive response: when site managers transform social media from a mere promotional tool into a flow management platform, its positive effects become significant. Data indicate that 35.2% of tourists are willing to adjust itineraries based on social media information, demonstrating that appropriate digital intervention can promote sustainable development at cultural heritage sites.

Path analysis results reveal a dual-pathway mechanism through which social media influences tourist response behavior in heritage tourism contexts. The cognitive-informational pathway operates primarily through decision confidence as a key mediating mechanism. Perceived usefulness demonstrated the strongest total effect on tourist response willingness (β = 0.452, *p* < 0.001 direct; β = 0.312, *p* < 0.001 to decision confidence), indicating that functional value dominates emotional appeals in heritage tourism contexts. This contrasts with Kim and Fesenmaier’s^19^ finding in leisure tourism where emotional factors showed stronger effects (β = 0.523), reflecting heritage tourists’ pragmatic concerns about crowding and time constraints. Information timeliness emerged as the second strongest predictor (β = 0.387, *p* < 0.001 direct; β = 0.287, *p* < 0.001 to decision confidence), supporting Vu et al.‘s^36^ argument that real-time information creates competitive advantages, yet the substantial time lag identified in “Analysis of social media information and tourist flow status” (average 3.2-h delay) partially explains why this pathway translates to only 35.2% actual behavioral change, underscoring the critical role of information delivery timing^1^. Decision confidence itself significantly influences tourist response willingness (β = 0.221, *p* < 0.001), demonstrating that information quality attributes enhance behavioral intentions not only directly but also by strengthening tourists’ confidence in their adjustment decisions.

The affective-social pathway operates through emotional resonance as an alternative influence mechanism. Information source credibility (β = 0.268, *p* < 0.001) and platform interaction (β = 0.195, *p* < 0.01) both significantly predict emotional resonance, which in turn influences tourist response willingness (β = 0.183, *p* < 0.01). This pathway reveals a credibility-engagement synergy: while official accounts scored higher on credibility (4.12 vs. 3.68), KOL content achieves higher conversion rates through emotional storytelling and interactive engagement, consistent with Liu et al.‘s^40^ social media envy framework. The moderate effect sizes in this pathway suggest that functional complementarity between official accounts and KOLs represents a strategic imperative rather than a competitive choice. The negative moderating effect of past travel experience (β = −0.156, *p* < 0.05) indicates that experienced tourists demonstrate reduced susceptibility to social media guidance, supporting Fotis et al.‘s^37^ argument that digital influence operates most effectively on first-time visitors, though the relatively small effect size (ΔR^2^ = 0.024) suggests strategic intervention remains possible even for repeat visitors. These path coefficients collectively demonstrate that effective visitor flow management through social media requires integrating both cognitive and affective pathways rather than relying on single mechanisms, with the dominance of the cognitive-informational pathway (through decision confidence) over the affective-social pathway reflecting the unique demand for pragmatic, actionable information delivered at decision-critical moments in heritage tourism contexts.

Social media-based tourist flow management may contribute to multiple Sustainable Development Goals. Research results suggest that digital guidance strategies are associated with SDG 8.9 sustainable tourism objectives through their potential to optimize spatial-temporal distribution patterns^47^. This spatial-temporal optimization may reduce pressure on cultural heritage carrying capacity, potentially supporting SDG 11.4 cultural heritage protection objectives. While our study focuses on social media’s role in influencing tourist behavior, effective visitor flow management requires integrated strategies. For instance, at the Mogao Grottoes, management implements advance reservations, fixed time slots, and a maximum daily capacity of 6000 visitors, supplemented by real-time environmental monitoring (relative humidity 62%, CO₂ 1500 ppm thresholds) to trigger temporary closures when necessary. Such comprehensive approaches—combining physical capacity controls with digital information systems that may include social media channels—represent practical applications of sustainable visitor management aligned with SDG 11.4.

Social media monitoring systems within smart city frameworks provide innovative tools for SDG 12.b^48^. This study’s analysis of 100,000 social media posts demonstrates the feasibility of large-scale digital data collection and analysis for sustainable tourism assessment. The finding that 78.3% of tourists rely on social media for travel planning underscores the platform’s reach as a data source. Through techniques including LDA topic modeling (K = 8 topics) and BERT sentiment analysis (87.3% accuracy), we extracted structured information on tourist concerns, temporal patterns, and emotional responses to heritage site experiences. While this study focuses on understanding how social media information influences tourist behavior rather than establishing comprehensive monitoring systems, the analytical framework demonstrates how digital platforms may contribute to multi-dimensional tourism assessment—potentially including visitor sentiment analysis, behavioral pattern identification, and information dissemination effectiveness—supporting the development of data-driven management tools aligned with SDG 12.b.

Research findings provide heritage site managers with a social media-based flow control strategy framework. Experience from European destination management organizations demonstrates that publishing crowd alerts 2–3 h in advance is associated with itinerary adjustments among 35.2% of tourists^49^. Managers should establish coordination mechanisms between official accounts and KOLs, utilizing official channels to publish authoritative information while leveraging KOLs’ high engagement rates (8.7%) to expand dissemination reach. Based on social media strategy assessment frameworks^50^, sites need to construct a “monitoring-warning-guidance” three-tier response system: real-time monitoring of social media data to identify crowding trends, initiating tiered warnings when visitor flows reach 70% of carrying capacity, and guiding tourists toward secondary attractions through differentiated information delivery. Employee training is equally important, as frontline service personnel equipped with digital tools may be better positioned to support visitor experience quality^51^. This proactive management model transforms social media from a marketing tool into a precise flow control platform, achieving a win-win situation for visitor satisfaction and heritage conservation.

This study has several limitations requiring cautious interpretation of results. Sample selection was limited to five well-known cultural heritage sites, which may not fully represent the actual situation of small and medium-sized attractions, particularly areas with weak digital infrastructure. While 507 questionnaire samples meet statistical requirements, they remain insufficient relative to the annual total of 53.6 million visitors, and survey timing concentrated in specific months may introduce seasonal bias. Causal inference requires careful treatment, as unobserved variables may confound the relationship between social media influence and tourist behavioral change^52^. The use of cross-sectional data makes it difficult to capture dynamic change processes, and the long-term effects of social media strategies and tourist adaptive behaviors remain unclear. A key methodological limitation concerns the measurement of actual behavioral change (ABC). Although the study initially operationalized ABC as a construct, it was not included in the final structural model. This exclusion stems from the cross-sectional on-site sampling approach: respondents were surveyed at heritage destinations after they had already made their visit decisions and acted upon them. This methodology conflates behavioral intention (Tourist Response Willingness) with realized behavior, as participants’ expressed willingness to respond to social media guidance had likely already been translated into concrete actions (e.g., visit timing adjustments, site selection). Consequently, the traditional temporal distinction between behavioral intention and subsequent behavior becomes problematic in this research context, as willingness measures may capture post-hoc rationalization of behaviors already enacted rather than predictors of future actions. Future longitudinal research designs that survey tourists before and after their visits would better capture the intention-behavior relationship and allow for proper examination of ABC as a distinct outcome. Additionally, digital divide issues among different age groups were not fully considered, with only 31.6% of tourists over 50 using social media, requiring alternative solutions for flow management of this demographic. At the technical level, the opacity of social media algorithms and platform policy changes may affect the stability of information dissemination effectiveness.

## Conclusion

Through empirical analysis of five Chinese cultural heritage sites, this study examines the associations between social media marketing and tourist flow management. The research reveals that 78.3% of tourists rely on social media information for itinerary planning, with 67.4% adjusting visiting times based on real-time crowd information and 35.2% willing to travel off-peak to avoid crowds, suggesting the potential of digital guidance strategies. The authoritative information dissemination by official accounts (credibility score 4.12) and high-engagement communication by KOLs (engagement rate 8.7%) form complementary mechanisms, jointly promoting spatial-temporal tourist distribution. Although 65% of current crowd alert information suffers from time lag issues, the key influencing factors revealed by this study, such as “real-time crowds” and “queue time,” provide clear directions for strategy optimization. This social media-based management model may support progress toward SDGs 8.9, 11.4, and 12.b, offering a Chinese approach to addressing overtourism challenges. Future research should focus on improving information publication timeliness, evaluating long-term effects, and examining cross-cultural applicability, driving global cultural heritage sites toward intelligent and sustainable transformation.

## Supplementary Information

Below is the link to the electronic supplementary material.

Supplementary Material 1

Supplementary Material 2

## Data Availability

The original contributions presented in the study are included in the article Material, further inquiries can be directed to the corresponding authors.
