# Supplementary material for: Social media marketing and digital influence for visitor flow management in sustainable heritage tourism
Source: Sci Rep. 2025 Dec 11;15:45767. doi: 10.1038/s41598-025-28555-9 (PMC12756228; doi:10.1038/s41598-025-28555-9)
Supplement: Supplementary file 1 — Supplementary Material 1 [file 41598_2025_28555_MOESM1_ESM.docx]

**Appendix A: Measurement Scales and Items**

All constructs were measured using a 5-point Likert scale (1 = Strongly Disagree, 5 = Strongly Agree) unless otherwise specified. Items were originally developed in English and translated into Chinese following the back-translation procedure to ensure semantic equivalence.

**I. Social Media Information Characteristics**

**1. Information Source Credibility (ISC)**

*Adapted from Chung & Han [18] and Xiang & Gretzel [16]*

| **Code** | **Item** |
| --- | --- |
| ISC1 | The social media accounts I follow provide reliable information about heritage sites. |
| ISC2 | I trust the crowd alert information published by official heritage site accounts. |
| ISC3 | The visitor flow information from social media sources is accurate and trustworthy. |
| ISC4 | Social media influencers (KOLs) provide credible recommendations about visiting times. |

**2. Perceived Usefulness (PU)**

*Adapted from Kim & Fesenmaier [19] and Leung et al. [17]*

| **Code** | **Item** |
| --- | --- |
| PU1 | Social media information helps me plan my visit to heritage sites more effectively. |
| PU2 | Real-time crowd information on social media is useful for adjusting my itinerary. |
| PU3 | Social media posts help me avoid overcrowded periods at heritage attractions. |
| PU4 | Information about waiting times and queue lengths on social media improves my travel decisions. |
| PU5 | Social media recommendations enable me to choose optimal visiting times. |

**3. Information Timeliness (IT)**

*Adapted from Fotis et al. [31] and Vu et al. [30]*

| **Code** | **Item** |
| --- | --- |
| IT1 | The crowd alert information I receive on social media is timely. |
| IT2 | Social media provides real-time updates about visitor flows at heritage sites. |
| IT3 | I can obtain up-to-date information about site crowding levels through social media. |
| IT4 | Social media platforms deliver visitor flow information faster than other channels. |

**4. Platform Interaction (PI)**

*Adapted from Whiting & Williams [36] and Liu et al. [37]*

| **Code** | **Item** |
| --- | --- |
| PI1 | I frequently engage with (like, comment, share) social media posts about heritage tourism. |
| PI2 | I actively participate in discussions about visiting experiences on social media platforms. |
| PI3 | I often interact with other users' posts regarding heritage site recommendations. |
| PI4 | Social media platforms facilitate my communication with other tourists about travel planning. |

**5. Decision Confidence (DC)**

*Adapted from Chung & Han [18] and Fotis et al. [31]*

| **Code** | **Item** |
| --- | --- |
| DC1 | I feel confident in adjusting my visiting time based on social media information about heritage sites. |
| DC2 | Social media information gives me confidence in making decisions to avoid overcrowded periods. |
| DC3 | I am certain that my travel planning decisions based on social media are appropriate. |
| DC4 | I trust my judgment when using social media information to optimize my heritage site visits. |

**6. Emotional Resonance (ER)**

*Adapted from Liu et al. [37] and Chung & Han [18]*

| **Code** | **Item** |
| --- | --- |
| ER1 | Social media posts about heritage sites evoke emotional responses in me. |
| ER2 | KOL content about cultural tourism creates a sense of connection with me. |
| ER3 | Visual content (photos/videos) on social media inspires my desire to visit heritage sites. |
| ER4 | Stories shared by other tourists on social media resonate with my travel aspirations. |

**II. Tourist Response and Behavior**

**7. Tourist Response Willingness (TRW)**

*Adapted from Ajzen [6] (Theory of Planned Behavior) and Chung & Han [18]*

| **Code** | **Item** |
| --- | --- |
| TRW1 | I am willing to adjust my visiting time based on crowd information from social media. |
| TRW2 | I would consider visiting heritage sites during off-peak periods if recommended on social media. |
| TRW3 | I am likely to change my travel plans to avoid overcrowding based on social media alerts. |
| TRW4 | I would accept alternative site recommendations provided through social media. |
| TRW5 | I intend to follow social media guidance for visitor flow management. |

**8. Actual Behavioral Change (ABC)**

*Adapted from Kim & Fesenmaier [19] and Mihalic [10]*

| **Code** | **Item** |
| --- | --- |
| ABC1 | I have previously adjusted my visiting time after seeing crowd alerts on social media. |
| ABC2 | I have changed my travel itinerary based on real-time information from social media. |
| ABC3 | I have chosen to visit heritage sites during recommended off-peak hours. |
| ABC4 | I have avoided certain dates/times at heritage attractions due to social media warnings. |

**III. Social Media Usage Patterns**

**9. Social Media Usage Intensity (SMUI)**

*Self-developed based on Gretzel [1] and Song & Abukhalifeh [11]*

| **Code** | **Item** | **Scale Type** |
| --- | --- | --- |
| SMUI1 | How frequently do you use social media for travel planning? | 1=Never to 5=Very Frequently |
| SMUI2 | How much time do you spend browsing heritage tourism content on social media daily? | 1=Less than 15 min to 5=More than 2 hours |
| SMUI3 | How many social media platforms do you use to search for heritage site information? | Open-ended (numerical) |

**10. Platform Usage (PU_Platform)**

*Multiple choice question*

**Question:** Which social media platforms do you primarily use to obtain heritage tourism information? (Select all that apply)

Weibo

TikTok (Douyin)

Rednote (Xiaohongshu)

WeChat

Other (please specify): _______

**IV. Demographic and Control Variables**

**11. Demographics**

| **Variable** | **Question** | **Response Options** |
| --- | --- | --- |
| Age | What is your age group? | 1=18-25; 2=26-35; 3=36-45; 4=46-55; 5=56+ |
| Gender | What is your gender? | 1=Male; 2=Female; 3=Other |
| Education | What is your highest education level? | 1=High school or below; 2=Bachelor's; 3=Master's; 4=Doctoral |
| Visit Frequency | How many times have you visited this heritage site? | 1=First time; 2=2-3 times; 3=4-5 times; 4=More than 5 times |
| Travel Party | Who are you traveling with? | 1=Alone; 2=Family; 3=Friends; 4=Tour group |

**12. Past Travel Experience (Control Variable)**

| **Code** | **Item** |
| --- | --- |
| PTE1 | I have extensive experience visiting cultural heritage sites in China. |
| PTE2 | I am familiar with the characteristics and visiting requirements of major heritage sites. |
| PTE3 | I usually plan my heritage tourism trips independently without relying heavily on external information. |

**V. Open-Ended Questions**

**OE1:** What factors most influence your decision to adjust visiting times at heritage sites? (Please rank top 3)

**OE2:** Have you ever experienced overcrowding at heritage sites? If yes, how did it affect your experience?

**OE3:** What type of social media information would be most helpful for planning your visit to cultural heritage sites?

**Notes:**

1. **Reverse-coded items:** None in the final version (removed during pilot testing to reduce respondent confusion).
2. **Questionnaire structure:**
   - Section 1: Social Media Usage (SMUI, Platform Usage)
   - Section 2: Information Characteristics (ISC, PU, IT, PI, ER)
   - Section 3: Behavioral Intentions and Actions (TRW, ABC)
   - Section 4: Demographics and Controls
   - Section 5: Open-ended questions
3. **Pilot testing:** The questionnaire was pilot-tested with 50 respondents in March 2024 to ensure clarity and reliability before full-scale distribution.
4. **Language:** The questionnaire was administered in Simplified Chinese. English translations are provided here for international readership.

**Construct Definitions**

| **Construct** | **Operational Definition** |
| --- | --- |
| **Information Source Credibility** | The degree to which tourists perceive social media information sources (official accounts and KOLs) as trustworthy and reliable. |
| **Perceived Usefulness** | Tourists' subjective assessment of how social media information helps them make better travel decisions and avoid overcrowding. |
| **Information Timeliness** | The extent to which social media information is delivered in a timely manner to enable proactive trip adjustments. |
| **Platform Interaction** | The frequency and intensity of tourists' engagement with social media content related to heritage tourism. |
| **Emotional Resonance** | The emotional connection and inspiration tourists feel from social media content about heritage sites. |
| **Tourist Response Willingness** | Tourists' intention to adjust their visiting behavior based on social media information. |
| **Actual Behavioral Change** | The extent to which tourists have actually modified their travel plans due to social media influence. |
| **Past Travel Experience** | Tourists' accumulated experience with heritage site visitation, which may moderate their reliance on social media. |
