# Supplementary material for: Social media marketing and digital influence for visitor flow management in sustainable heritage tourism
Source: Sci Rep. 2025 Dec 11;15:45767. doi: 10.1038/s41598-025-28555-9 (PMC12756228; doi:10.1038/s41598-025-28555-9)
Supplement: Supplementary file 2 — Supplementary Material 2 [file 41598_2025_28555_MOESM2_ESM.docx]

**Appendix B:**

This appendix provides supplementary data supporting the main analyses reported in the manuscript. Tables S1-S3 present descriptive statistics from the questionnaire survey, while Tables S4-S7 report comprehensive psychometric assessments of the measurement model used in the Partial Least Squares Structural Equation Modeling (PLS-SEM) analysis.

**Table S1: Questionnaire Survey Overview and Tourist Social Media Usage Characteristics**

| **Survey Indicator** | **Value** | **Note** |
| --- | --- | --- |
| **Questionnaire Distribution and Collection** |  |  |
| **Total questionnaires distributed** | 653 | Across five heritage sites |
| **Incomplete questionnaires** | 146 | Missing key information or uncompleted |
| **Valid questionnaires** | 507 | Used for final analysis |
| **Effective response rate** | 77.6% | 507/653 |
| **Social Media Information Seeking Behavior** |  |  |
| **Actively search for social media information before travel** | 78.3% | 397 tourists |
| **Do not actively search** | 21.7% | 110 tourists |

**Table S2: Distribution of Tourist Social Media Platform Usage Preferences**

| **Platform Type** | **Usage Proportion** | **Sample Size** | **Main Content Characteristics** |
| --- | --- | --- | --- |
| **Rednote** | 42.5% | 216 | Image-text guides, recommendations |
| **TikTok (Douyin)** | 38.7% | 196 | Short videos, influencer check-ins |
| **Weibo** | 18.8% | 95 | Real-time updates, official announcements |

*Note: Respondents could select multiple platforms; percentages reflect primary platform usage.*

**Table S3: Differences in Social Media Dependence Across Age Groups**

| **Age Group** | **Social Media Information Dependence** | **Sample Size (n)** | **Percentage of Total Sample** | **Chi-square Test** |
| --- | --- | --- | --- | --- |
| **18-25 years** | 92.0% | 150 | 29.6% | χ²=156.34 |
| **26-35 years** | 86.1% | 137 | 27.0% | p<0.001 |
| **36-45 years** | 62.4% | 85 | 16.8% | df=4 |
| **46-55 years** | 47.4% | 57 | 11.2% |  |
| **56 years and above** | 31.6% | 78 | 15.4% |  |
| **Total** | 78.3% | 507 | 100.0% |  |

*Note: Social media information dependence refers to tourists who rely on social media as a primary source for travel planning. Chi-square test indicates significant differences across age groups (χ²=156.34, df=4, p<0.001).*

**Table S4: Reliability and Convergent Validity Assessment**

| **Construct** | **No. of Items** | **Cronbach's Alpha (α)** | **Composite Reliability (CR)** | **Average Variance Extracted (AVE)** |
| --- | --- | --- | --- | --- |
| Information Source Credibility (ISC) | 4 | 0.856 | 0.903 | 0.700 |
| Perceived Usefulness (PU) | 5 | 0.892 | 0.921 | 0.702 |
| Information Timeliness (IT) | 4 | 0.871 | 0.912 | 0.722 |
| Platform Interaction (PI) | 4 | 0.823 | 0.883 | 0.654 |
| Decision Confidence (DC) | 4 | 0.879 | 0.916 | 0.732 |
| Emotional Resonance (ER) | 4 | 0.847 | 0.897 | 0.685 |
| Tourist Response Willingness (TRW) | 5 | 0.908 | 0.932 | 0.733 |
| Past Travel Experience (PTE) | 3 | 0.794 | 0.879 | 0.708 |

*Note: All constructs meet the recommended thresholds: α > 0.70, CR > 0.70, AVE > 0.50.*

**Table S5: Factor Loadings and Cross-Loadings**

| **Item** | **ISC** | **PU** | **IT** | **PI** | **DC** | **ER** | **TRW** | **PTE** |
| --- | --- | --- | --- | --- | --- | --- | --- | --- |
| **ISC1** | 0.821 | 0.412 | 0.398 | 0.321 | 0.345 | 0.356 | 0.389 | 0.267 |
| **ISC2** | 0.867 | 0.451 | 0.423 | 0.298 | 0.367 | 0.387 | 0.412 | 0.289 |
| **ISC3** | 0.849 | 0.468 | 0.445 | 0.312 | 0.378 | 0.401 | 0.398 | 0.298 |
| **ISC4** | 0.795 | 0.391 | 0.367 | 0.345 | 0.334 | 0.378 | 0.356 | 0.243 |
| **PU1** | 0.438 | 0.812 | 0.523 | 0.389 | 0.567 | 0.421 | 0.587 | 0.334 |
| **PU2** | 0.456 | 0.854 | 0.567 | 0.412 | 0.589 | 0.445 | 0.612 | 0.312 |
| **PU3** | 0.421 | 0.867 | 0.543 | 0.398 | 0.578 | 0.432 | 0.598 | 0.298 |
| **PU4** | 0.398 | 0.823 | 0.512 | 0.378 | 0.556 | 0.411 | 0.567 | 0.287 |
| **PU5** | 0.412 | 0.841 | 0.534 | 0.391 | 0.571 | 0.423 | 0.589 | 0.301 |
| **IT1** | 0.423 | 0.534 | 0.856 | 0.367 | 0.523 | 0.398 | 0.512 | 0.289 |
| **IT2** | 0.445 | 0.567 | 0.879 | 0.389 | 0.545 | 0.421 | 0.534 | 0.312 |
| **IT3** | 0.412 | 0.543 | 0.867 | 0.378 | 0.534 | 0.412 | 0.523 | 0.298 |
| **IT4** | 0.398 | 0.521 | 0.789 | 0.356 | 0.512 | 0.389 | 0.498 | 0.276 |
| **PI1** | 0.321 | 0.389 | 0.367 | 0.823 | 0.334 | 0.456 | 0.378 | 0.298 |
| **PI2** | 0.334 | 0.412 | 0.389 | 0.845 | 0.356 | 0.478 | 0.398 | 0.312 |
| **PI3** | 0.298 | 0.378 | 0.356 | 0.791 | 0.323 | 0.445 | 0.367 | 0.287 |
| **PI4** | 0.312 | 0.398 | 0.378 | 0.776 | 0.334 | 0.423 | 0.356 | 0.276 |
| **DC1** | 0.345 | 0.567 | 0.523 | 0.334 | 0.856 | 0.378 | 0.612 | 0.312 |
| **DC2** | 0.367 | 0.589 | 0.545 | 0.356 | 0.871 | 0.398 | 0.634 | 0.334 |
| **DC3** | 0.378 | 0.578 | 0.534 | 0.323 | 0.867 | 0.389 | 0.623 | 0.323 |
| **DC4** | 0.334 | 0.556 | 0.512 | 0.334 | 0.828 | 0.367 | 0.598 | 0.298 |
| **ER1** | 0.378 | 0.432 | 0.398 | 0.456 | 0.378 | 0.834 | 0.423 | 0.298 |
| **ER2** | 0.401 | 0.445 | 0.421 | 0.478 | 0.398 | 0.856 | 0.445 | 0.312 |
| **ER3** | 0.389 | 0.423 | 0.412 | 0.445 | 0.389 | 0.821 | 0.434 | 0.301 |
| **ER4** | 0.367 | 0.411 | 0.389 | 0.423 | 0.367 | 0.798 | 0.412 | 0.287 |
| **TRW1** | 0.398 | 0.589 | 0.523 | 0.378 | 0.612 | 0.434 | 0.847 | 0.334 |
| **TRW2** | 0.412 | 0.612 | 0.534 | 0.398 | 0.634 | 0.445 | 0.871 | 0.356 |
| **TRW3** | 0.389 | 0.598 | 0.512 | 0.367 | 0.623 | 0.423 | 0.865 | 0.345 |
| **TRW4** | 0.367 | 0.567 | 0.498 | 0.356 | 0.598 | 0.412 | 0.823 | 0.323 |
| **TRW5** | 0.378 | 0.587 | 0.523 | 0.378 | 0.612 | 0.434 | 0.856 | 0.338 |
| **PTE1** | 0.276 | 0.312 | 0.298 | 0.298 | 0.312 | 0.301 | 0.345 | 0.867 |
| **PTE2** | 0.289 | 0.334 | 0.312 | 0.312 | 0.334 | 0.312 | 0.356 | 0.851 |
| **PTE3** | 0.254 | 0.287 | 0.276 | 0.287 | 0.298 | 0.287 | 0.323 | 0.803 |

*Note: Bolded values represent factor loadings on respective constructs. All loadings > 0.70 indicate adequate convergent validity. Cross-loadings are substantially lower than main loadings, supporting discriminant validity.*

**Table S6: Discriminant Validity Assessment (HTMT Ratios)**

|  | **ISC** | **PU** | **IT** | **PI** | **DC** | **ER** | **TRW** | **PTE** |
| --- | --- | --- | --- | --- | --- | --- | --- | --- |
| **ISC** | - |  |  |  |  |  |  |  |
| **PU** | 0.542 | - |  |  |  |  |  |  |
| **IT** | 0.498 | 0.687 | - |  |  |  |  |  |
| **PI** | 0.412 | 0.487 | 0.456 | - |  |  |  |  |
| **DC** | 0.456 | 0.723 | 0.654 | 0.423 | - |  |  |  |
| **ER** | 0.476 | 0.534 | 0.498 | 0.612 | 0.487 | - |  |  |
| **TRW** | 0.487 | 0.743 | 0.634 | 0.465 | 0.768 | 0.543 | - |  |
| **PTE** | 0.356 | 0.398 | 0.376 | 0.389 | 0.412 | 0.398 | 0.423 | - |

*Note: All HTMT values < 0.85, indicating adequate discriminant validity. Values < 0.90 are considered acceptable in exploratory research.*

**Table S7: Collinearity Assessment (VIF Values)**

| **Construct** | **VIF (Inner Model)** | **Assessment** |
| --- | --- | --- |
| Perceived Usefulness → Decision Confidence | 1.687 | No collinearity issue |
| Information Timeliness → Decision Confidence | 1.745 | No collinearity issue |
| Decision Confidence → Tourist Response Willingness | 2.234 | No collinearity issue |
| Perceived Usefulness → Tourist Response Willingness | 2.456 | No collinearity issue |
| Information Timeliness → Tourist Response Willingness | 2.187 | No collinearity issue |
| Information Source Credibility → Emotional Resonance | 1.654 | No collinearity issue |
| Platform Interaction → Emotional Resonance | 1.543 | No collinearity issue |
| Emotional Resonance → Tourist Response Willingness | 1.876 | No collinearity issue |
| Past Travel Experience × Tourist Response Willingness (Moderator) | 1.432 | No collinearity issue |

*Note: All VIF values < 3.0, indicating no multicollinearity concerns. Threshold: VIF < 5.0 (conservative: < 3.0).*
